# Whether sutures reduce the graft laceration caused by interference screw in anterior cruciate ligament reconstruction? A biomechanical study in vitro

**DOI:** 10.1186/s12891-021-04457-5

**Published:** 2021-06-22

**Authors:** Yuanjun Teng, Xiaohui Zhang, Lijun Da, Jie Hu, Hong Wang, Hua Han, Meng Wu, Shifeng Zhang, Yayi Xia

**Affiliations:** 1 Department of Orthopaedics, Lanzhou University Second Hospital, Lanzhou University, No. 82 Cuiyingmen, Chengguan District, 730030 Lanzhou City, Gansu Province People’s Republic of China; 2 Orthopaedics Key Laboratory of Gansu Province, Lanzhou University Second Hospital, Lanzhou University, 730030 Lanzhou City, Gansu Province People’s Republic of China; 3 Department of Oncology, Lanzhou University Second Hospital, Lanzhou University, 730030 Lanzhou City, Gansu Province People’s Republic of China; 4 The Second Clinical Medical College, Lanzhou University, 730030 Lanzhou City, Gansu Province People’s Republic of China

**Keywords:** Graft laceration, Interference screws, Suture, Anterior cruciate ligament reconstruction, Biomechanical study

## Abstract

**Background:**

Interference screw is commonly used for graft fixation in anterior cruciate ligament (ACL) reconstruction. However, previous studies had reported that the insertion of interference screws significantly caused graft laceration. The purposes of this study were to (1) quantitatively evaluate the graft laceration from one single insertion of PEEK interference screws; and (2) determine whether different types of sutures reduced the graft laceration after one single insertion of interference screws in ACL reconstruction.

**Methods:**

The in-vitro ACL reconstruction model was created using porcine tibias and bovine extensor digitorum tendons of bovine hind limbs. The ends of grafts were sutured using three different sutures, including the bioabsorbable, Ethibond and ultra-high molecular weight polyethylene (UHMWPE) sutures. Poly-ether-ether-ketone (PEEK) interference screws were used for tibial fixation. This study was divided into five groups (*n* = 10 in each group): the non-fixed group, the non-sutured group, the absorbable suture group, the Ethibond suture group and the UHMWPE suture group. Biomechanical tests were performed using the mode of pull-to-failure loading tests at 10 mm/min. Tensile stiffness (newtons per millimeter), energy absorbed to failure (in joules) and ultimate load (newtons) were recorded for analysis.

**Results:**

All prepared tendons and bone specimens showed similar characteristics (length, weight, and pre-tension of the tendons, tibial bone mineral density) among all groups (*P >* 0.05). The biomechanical tests demonstrated that PEEK interference screws significantly caused the graft laceration (*P <* 0.05). However, all sutures (the bioabsorbable, Ethibond and UHMWPE sutures) did not reduce the graft laceration in ACL reconstruction (*P* > 0.05).

**Conclusions:**

Our biomechanical study suggested that the ultimate failure load of grafts was reduced of approximately 25 % after one single insertion of a PEEK interference screw in ACL reconstruction. Suturing the ends of the grafts using different sutures (absorbable, Ethibond and UHMWPE sutures) did not decrease the graft laceration caused by interference screws.

## Introduction

Anterior cruciate ligament (ACL) plays an essential role in knee stability. ACL deficiency might cause meniscus tear and articular cartilage degeneration, which seriously affects the function of the knee joint [1]. The goals of ACL reconstruction are to increase the functional stability of the knee joint and decrease secondary damage to meniscus and articular cartilage [2–4]. However, a high revision rate has been reported after primary ACL reconstructions [5–8]. A register-based study including 38,666 patients showed that the 5-year revision rate of using the interference screw was 4.2 % [7]. Data from the Swedish register center on 13,102 Patients showed that the overall 2-year ACL revision rate was 1.60 % (female, 1.57 %; male, 1.63 %) [6].Hamstring tendon autograft has become an increasingly popular choice for ACL reconstruction [9, 10]. Interference screw is widely accepted for graft fixation, which provides direct fixation by compressing the graft against the wall of the tibial tunnel and improves the osteointegration of graft and bone [8, 11]. However, significant concerns regarding the usage of interference screws are graft laceration and the substantial loss of fixation strength [12]. Zantop et al. [13] reported that interference screw insertion leaded to a macroscopic damage to ACL graft. Correspondingly, several fixation methods are devised to decrease graft damage, such as the sheath equipment outside the screw [14] or the use of expandable interference screw [15].

During ACL reconstruction, suturing the free ends of ACL grafts in a whipstitch fashion is a simple procedure, which helps surgeons to handle the graft and maintain an equal tension among the graft strands [16]. Previous studies had demonstrated that suturing the free ends of the graft significantly affected the biomechanical properties of graft-bone complex [17, 18]. The related mechanism was that sutures improved the engagement of the interference screw and grafts, which resulted in better fixation strength within the tibial tunnel [18, 19]. Another possible mechanism was that sutures used for suturing the ends of grafts provide protective effects on grafts, which reduces tendon fiber damage from the screw insertion and keeps the integrity of graft. However, this hypothesis has not been validated.

Therefore, we designed this study to (1) quantitatively evaluate the graft laceration from one single insertion of PEEK interference screws; and (2) determine whether different types of sutures reduced the graft laceration after one single insertion of interference screws in ACL reconstruction. We hypothesized that the insertion of PEEK interference screws significantly caused the graft laceration, and sutures might decrease tendon damage during tibial fixation.

## Methods

This study protocol was approved by the ethics committee of our hospital.

### Specimens and study groups

Fresh-frozen porcine tibias (mean age, 12 ± 2.1 months) and bovine extensor digitorum tendons of bovine hind limbs (mean age, 25 ± 3.2 months) were used in our study. Specimens were obtained from a laboratory animal center. Porcine tibias have shown similar biomechanical properties with the adult human knees [14, 20]. And bovine extensor tendons have similar viscoelastic, structural and material properties to human tendons [21, 22]. Both were stored at -20 ° C. The grafts were randomly divided into five groups: the non-fixed group (without screw intervention, *n* = 10), the non-sutured group (screw intervention, *n* = 10), the absorbable suture group (absorbable suture + screw intervention, *n* = 10), the Ethibond suture group (Ethibond suture + screw intervention, *n* = 10) and the UHMWPE suture group (UHMWPE suture + screw intervention, *n* = 10). Three kinds of suturing materials were used in the suture groups: bioabsorbable suture [VICRYL Plus, 2.0, ETHICON Inc.], Ethibond suture [ETHIBOND EXCEL, 2.0, ETHICON Inc.] and ultra-high molecular weight polyethylene suture [UHMWPE, 2.0, Arthrex Inc.]. All sutures were of standard Pharmacopeia size (No. 2).

### Graft and tibial tunnel preparations

**Fig. 1:**
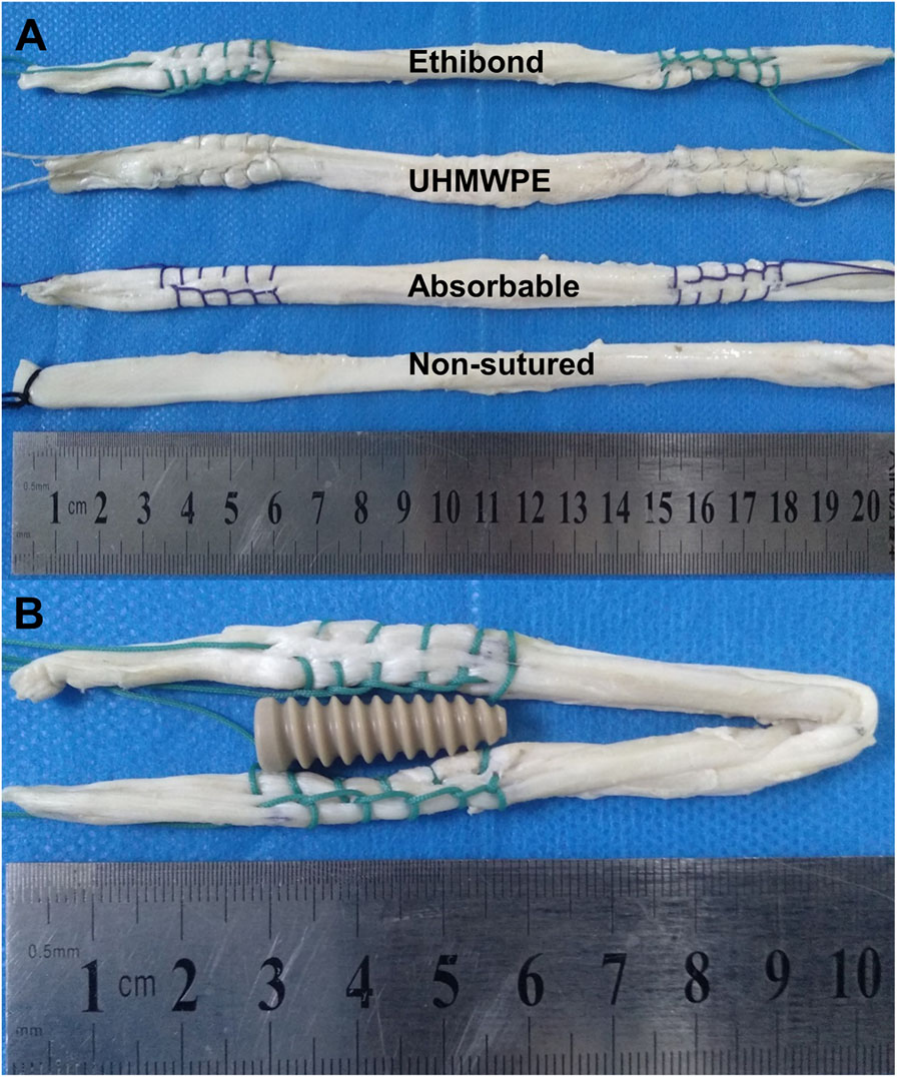
Preparations for grafts: **A**. the prepared graft specimens in single strand with 20 cm in length; **B**. an example of the Ethibond sutured graft specimens in double strands with 8.0 mm in diameter and 10 cm in length, the middle part of graft was sutured for 30 mm for the fixation of interference screw

The tibias and tendons were thawed to room temperature 12 h before specimen making. A single-stranded tendon was trimmed to a length of 200 mm (Fig. 1A), and then folded to produce a double-stranded graft with 8.0 mm in diameter and 100 mm in length (Fig. 1B). Tendons were weighed to obtain similar characteristics for grafts. The length included 30 mm for free from the end of the graft and 30 mm for screw fixation. In the three suture groups, the ends of grafts were sutured with an absorbable, Ethibond, or UHMWPE suture in a standard whipstitch fashion [stitch number (*n* = 6), length (30 mm)]. Then grafts were wrapped in 0.9 % saline-soaked gauze until use.

After graft preparation, a porcine tibia was matched with graft to undergo the following preparation. A 2.0 mm Kirschner wire was inserted in a site that located in the medial 1 cm of tibial tubercle, approximately 3 cm below the tibia plateau, to the center of ACL tibial footprint guided by a 55° ACL device [23, 24]. Then, a tibial tunnel with 8 mm in dimeter was created along the guide pin. The bone mineral density (BMD) of tibias was measured using dual-energy X-ray absorptiometry to eliminate the impact of BMD on biomechanical tests.

### Tbial fixation techniques

A poly-ether-ether-ketone (PEEK) (Arthrex Inc., USA) interference screw (8mm in diameter and 28 mm in length) was used for tibial fixation in this study. In the non-fixed group, grafts received no intervention (the blank control group). In the non-sutured group, grafts were not sutured but received one single insertion of a PEEK interference screw. In the suture groups, the ends of grafts were sutured using an absorbable, or UHMWPE suture, and then received the intervention with an interference screw. To achieve a central fixation of the screw, an interference screw was inserted between the graft and the tunnel along a guide wire until the screw tail aligned with anterior tibial cortex [25]. After screw insertion, the tibia was split with a manual saw, and the graft was carefully retrieved. This ensured that our study was consistent with the actual clinical practice, because the graft was only damaged once by one single insertion of screw. Lastly, sutures were carefully removed. Above procedures were completed by one surgeon team collaboratively.

### Biomechanical test

**Fig. 2:**
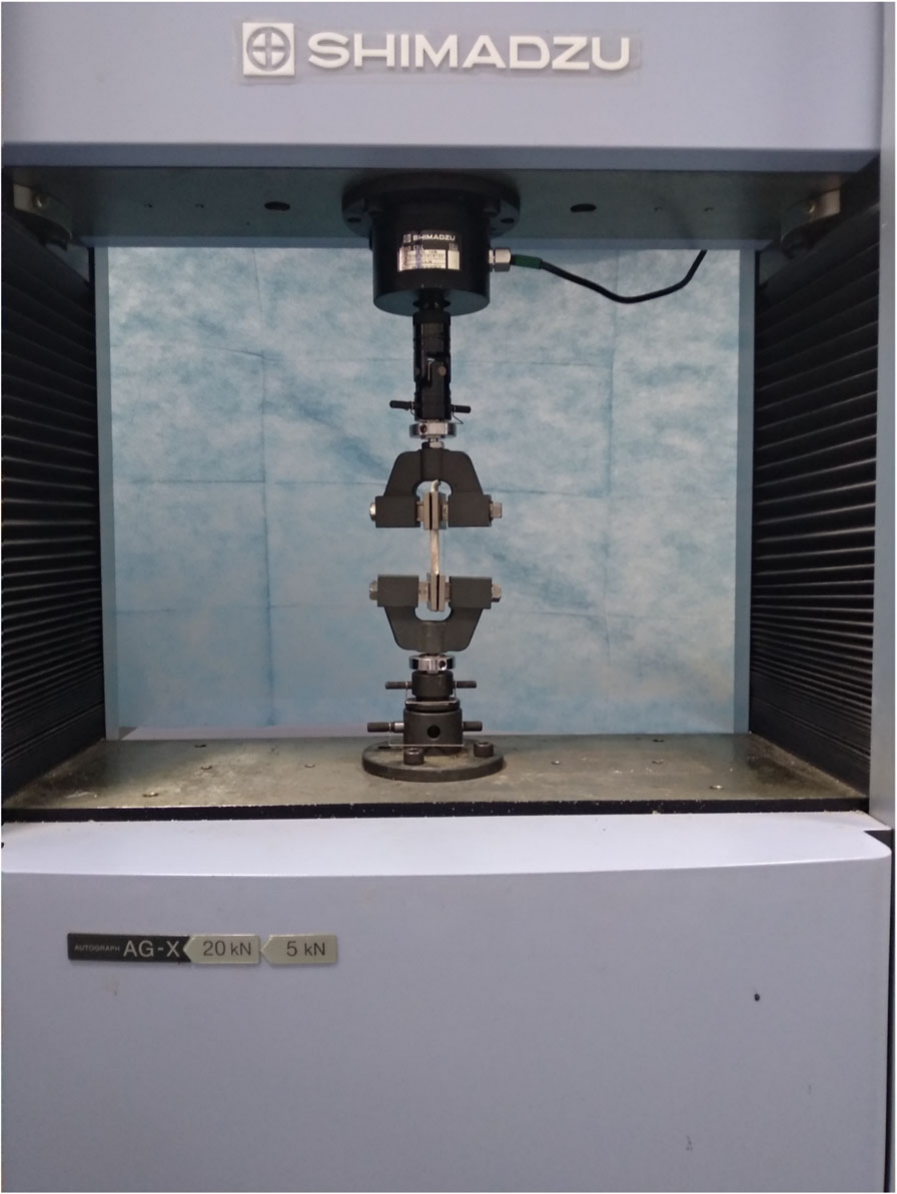
The biomechanical test for evaluation of graft laceration

**Fig. 3:**
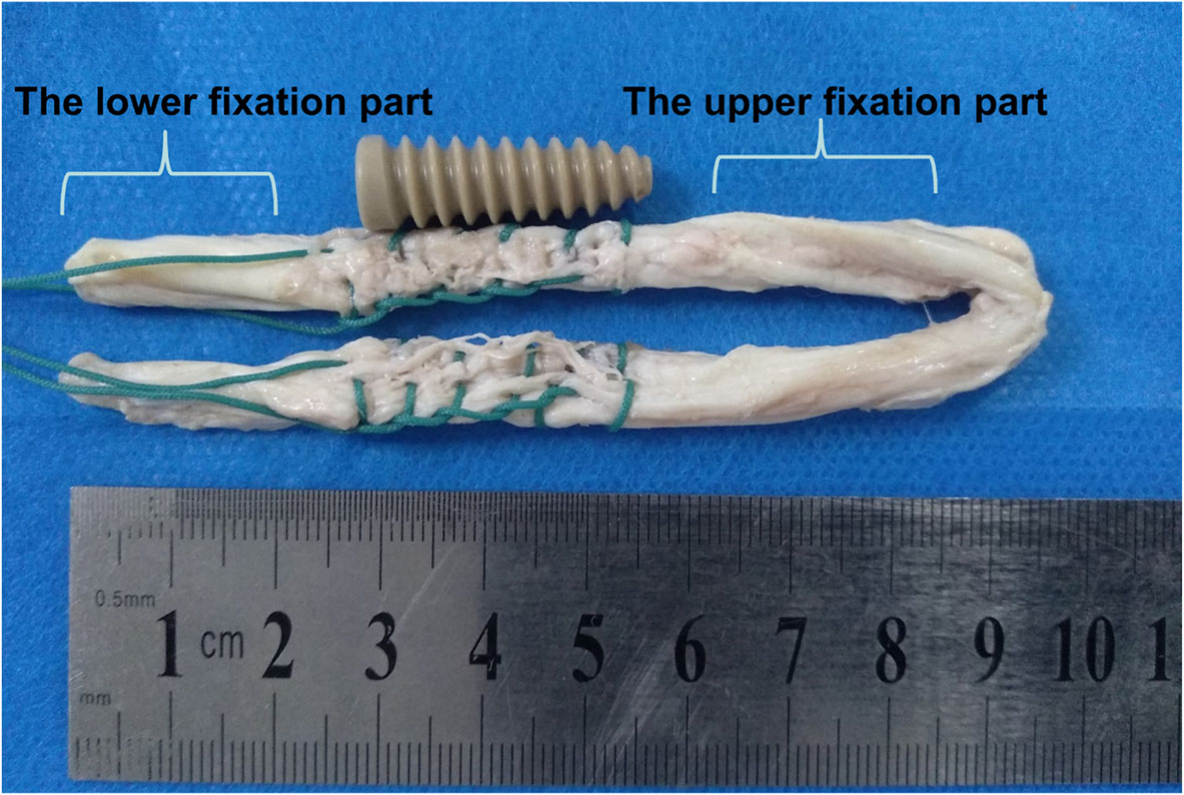
The fixation parts for the upper and lower clamps in the biomechanical test

The graft was taken out for a biomechanical test using an electronic universal testing machine (Shimadzu, AG-X series vertical machine, Japan). Graft laceration and protective effects of sutures on grafts were assessed by pull-to-failure loading tests as previous studies reported [13] (Fig. 2). The fixation length of upper and lower parts for the clamps is about 20 mm. The end of fixation part is about 5 mm to the sutured grafts (Fig. 3). We used a software (Trapezium X, Shimadzu Limited, Japan) for data collection. All grafts were preloaded from 10 to 50 N at a frequency of 0.1 Hz for 10 cycles [20, 26]. Subsequently, grafts were loaded at 10 mm/min to failure. Tensile stiffness (N/mm), energy at failure (J), the ultimate load (N) and the mode of graft failure (graft rupture or slippage, and the site of graft rupture) were recorded. Stiffness was calculated from the raw data of the load-elongation curve.

### Statistical analysis

All statistical analyses were performed with SPSS Statistics 22.0 (SPSS, Inc., Chicago, IL, USA). The mean differences between groups were assessed by one-way analysis of variance (ANOVA). *P* < 0.05 was used to determine statistical significance.

## Results

### The basic characteristics of the grafts and tibias

The porcine tibias and bovine grafts showed similar basic characteristics. There were no significant differences in graft length, graft weight and tibial BMD among groups (*P* > 0.05) (Table 1).

**Table 1:** Basic characteristics of the grafts and tibias

Basic characteristics	Non-fixed group	Non-sutured group	Bioabsorbable suture group	Ethibond suture group	UHMWPE suture group
Graft length (cm)^a^	20.33 ± 1.81	20.55 ± 1.13	20.58 ± 1.35	20.41 ± 1.51	20.28 ± 1.82
Graft weight (g)^a^	6.51 ± 1.21	6.93 ± 1.54	6.65 ± 1.82	6.75 ± 1.55	6.51 ± 1.72
Tibial BMD (g/cm^2^)^a^	1.25 ± 0.45	1.16 ± 0.49	1.30 ± 0.36	1.28 ± 0.41	1.18 ± 0.37

Above data are shown as means ± standard deviations

Abbreviation: *BMD* bone mineral density

^a^No differences were found among groups

### Outcomes of the biomechanical test

#### Graft laceration

In order to quantitatively investigate the graft laceration, the biomechanical comparisons were performed in the non-fixed and non-sutured groups. The results found that, after one single insertion of a PEEK interference screw, the ultimate failure load in the non-sutured group was significantly decreased by approximately 25 % than that in the non-fixed group (543.74 ± 101.78 N vs. 406.93 ± 108.42 N, *P* < 0.05). Regarding the failure mode, all grafts in the non-sutured and non-sutured groups were failed by graft rupture, and no grafts were failed by graft slippage. In the non-sutured group, the sites of graft rupture varied (3 in the upper fixation site, 5 in the mid-substance area, and 2 in the lower fixation site). In the non-sutured group, all grafts were ruptured in the insertion site of interference screws.

#### Protective effects of sutures on grafts

**Fig. 4:**
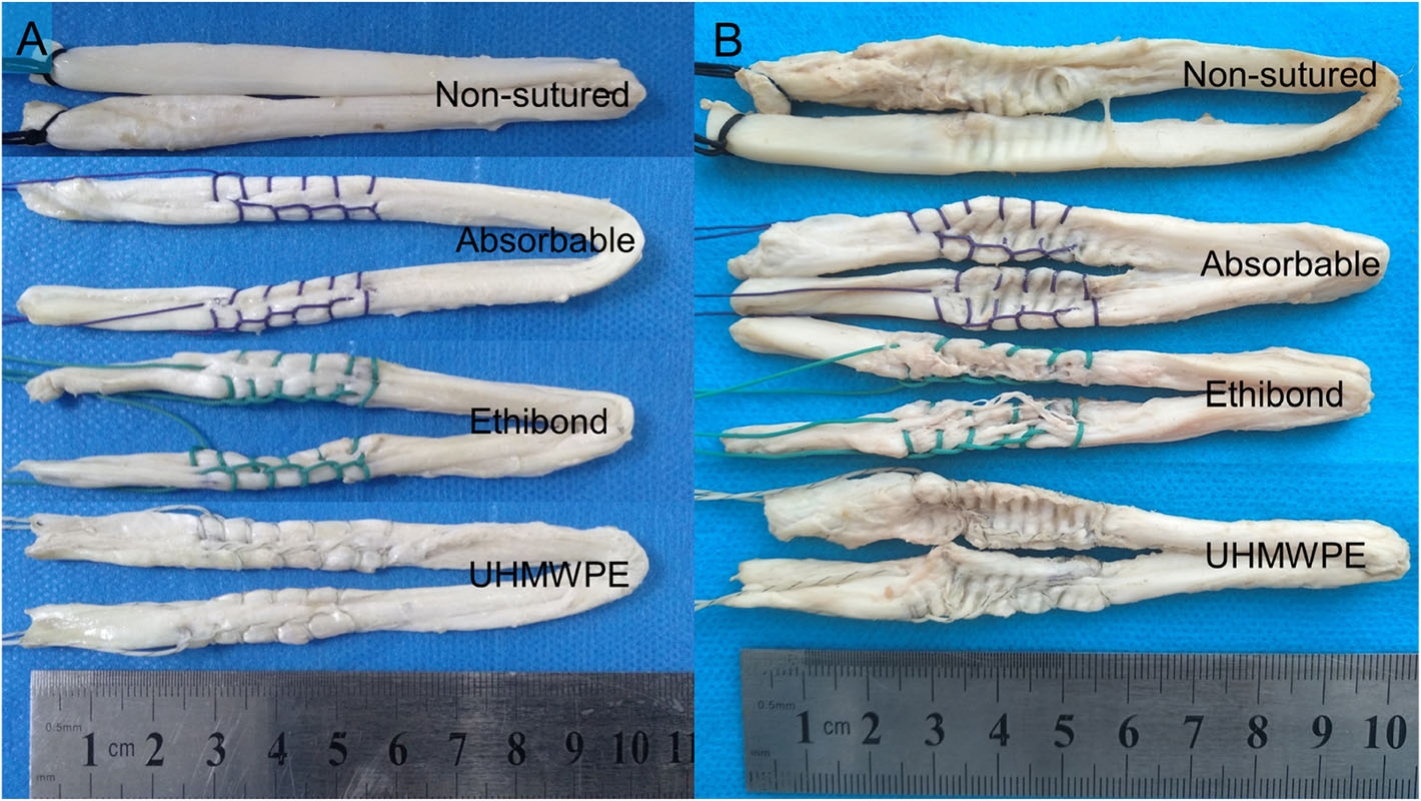
The comparison of grafts sutured using different sutures before and after one single insertion of a PEEK interference screw: **A**. before insertion of a PEEK interference screw; **B**. after insertion of a PEEK interference screw

**Fig. 5:**
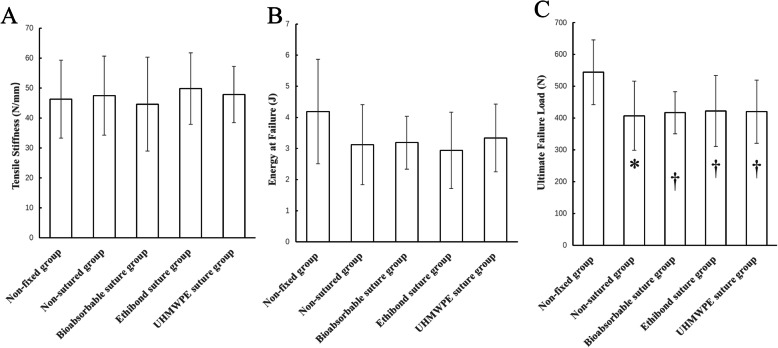
The biomechanical outcomes of in five groups: **A**. outcomes of tensile stiffness; **B**. outcomes of energy at failure; **C**. outcomes of ultimate failure load. * Compared with the non-fixed group, *P* < 0.05; † Compared with the non-sutured group, *p* > 0.05

The comparisons of the graft laceration before and after a PEEK interference screw insertion in each group were shown in Fig. 4. The biomechanical tests showed that the ultimate failure load was 406.93 ± 108.42 N in the non-sutured group, 416.61 ± 66.29 N in the bioabsorbable suture group, 422.08 ± 111.22 N in the Ethibond suture group, and 420.09 ± 99.32 N in the UHMWPE suture group respectively. Though the ultimate failure load was slightly increased in the suture groups, no statistical differences were found compared with the non-sutured group (*P >* 0.05). Moreover, no differences were found in terms of tensile stiffness and energy at failure (*P* > 0.05) among these 4 groups (Table 2; Fig. 5). Regarding the failure mode, all grafts in the bioabsorbable, Ethibond and UHMWPE suture groups were failed by graft rupture in the insertion site of interference screws.

**Table 2:** The biomechanical outcomes in five groups

Group	Tensile Stiffness (N/mm)	Energy at Failure(J)	Ultimate Failure Load(N)
Non-fixed group	46.34 ± 13.01	4.19 ± 1.68	543.74 ± 101.78
Non-sutured group	47.48 ± 13.22	3.12 ± 1.29	406.93 ± 108.42*
Bioabsorbable suture group	44.63 ± 15.69	3.19 ± 0.85	416.61 ± 66.29†
Ethibond suture group	49.83 ± 11.92	2.94 ± 1.23	422.08 ± 111.22†
UHMWPE suture group	47.83 ± 9.42	3.34 ± 1.09	420.09 ± 99.32†

Above data are shown as means ± standard deviations

*Compared with the non-fixed group, *P* < 0.05

†Compared with the non-sutured group, *p* > 0.05

## Discussion

This study was performed to determine whether sutures reduced graft laceration during tibial fixation in ACL reconstruction. The most important finding of this study was that sutures (absorbable, Ethibond and UHMWPE sutures) used for suturing ACL grafts in a whipstitch method did not provide protective effects on grafts.

The prognosis of primary ACL reconstruction is determined by multiple factors, including surgical techniques, methods of graft fixation, graft types and postoperative rehabilitation [8]. Among these, stable tibial fixation is essential for early postoperative rehabilitation [27]. Previous studies had reported that the tibial fixation methods significantly influenced the revision risk after ACL reconstruction [5, 7, 8]. Persson et al. [7] demonstrated that the 5-year revision rate was 4.2 %, 4.0 and 2.5 % for interference screw, Intrafix and Rigidfix, respectively. Eysturoy et al. [8] found that the interference screw fixation for a hamstring tendon showed the highest risk of revision during the follow-up period [8]. These findings raised concern regarding the applicationsof interference screws for ACL reconstruction.

Most biomechanical tests have shown that metallic or bioabsorbable interference screws provide sufficient fixation strength [28–30]. However, the insertion of interference screws during ACL reconstruction significantly leaded to graft laceration. Sawyer et al. [12] reported that interference screws weakened the biomechanical properties of the ACL graft, which resulted in less ultimate load, yield load and stiffness. Zantop et al. [13] demonstrated that the titanium interference screw may lead to more tendon damage in comparison with the biodegradable interference screws. The yield load of specimens in the biodegradable group (714.0 ± 333 N) was statistically higher than that of specimens in the titanium screw group (67.3 ± 44 N) (*P* < 0.05). In our study, we used PEEK interference screws for tibial fixation, and the outcomes showed that PEEK interference screws caused significant damage to ACL grafts. After one single insertion of a PEEK interference screw, the ultimate failure load of the ACL graft in the non-sutured group was significantly decreased by approximately 25 % compared with the non-fixed group. Therefore, surgeons should consider the tendon damage from screws while preparing the grafts.

During graft preparation, sutures are usually used for obtaining an even tensioning and equal distribution of forces between the strands of grafts while performing tibial fixation [16]. Moreover, sutures on the surface of grafts might decrease the cutting damage from the insertion of interference screws. In this study, we used three kinds of suturing materials for suturing the fixation part of the ACL grafts to investigate whether sutures provided a protective effect on grafts. For the measurement of graft laceration, we used an indirect method (a load-to-failure test) to quantitatively evaluate the graft damage as recommended by Zantop et al. [13]. As reported in other studies [19, 31, 32], the ultimate failure load was selected as the most representative value of fixation failure. Interestingly, our results demonstrated that, the grafts sutured using the bioabsorbable, Ethibond or UHMWPE suture showed similar ultimate failure load compared with that in the non-sutured group. Based on this result, our hypothesis that suturing the fixation part of the ACL graft decreased the graft laceration from the insertion of interference screws was not supported. One possible explanation was that, although different sutures were used for suturing the grafts, but the same suturing method (a whipstitch fashion) produced a similar interrupted space on the surfaces of tendon. The interference screw may damage tendon fiber through the interrupted space.

The bioabsorbable and Ethibond sutures are two of the most commonly used sutures for ACL graft preparation. Ethibond sutures are made of braided polyester with higher strength than bioabsorbable sutures. The UHMWPE suture is a new suture material, which has greater tensile strength than Ethibond sutures [33, 34]. Wright et al. reported that the biomechanical properties of the UHMWPE suture would be maintained even when they are partially damaged [35]. Despite UHMWPE sutures showed improved biomechanical properties, our results did not found that this suture significantly reduced graft laceration compared with the bioabsorbable and Ethibond sutures. One possible reason was that the material of UHMWPE sutures determined that it was more likely to slip than the other two sutures. Previous studies reported that knot slippage was more frequently occurred when knots tied using the UHMWPE sutures while it was under cyclic testing, and Ethibond sutures was the least likely to slip [33, 36].

During the ACL reconstruction, surgeons are more concerned about the fixation strength of ACL grafts. As previous studies [18, 19] had reported the effect of graft suturing on the pullout strength, we did not perform similar tests in our study. Prado et al. [18] demonstrated that suturing the graft may result in an increase in yield load of about 50 %. Hoher et al. [18] found that suturing of the graft construct significantly increased ultimate failure load by about 30 %, but superior outcomes was not confirmed in response to cyclic loading. Therefore, they concluded that, although suturing grafts improved the ultimate failure load of the graft construct, a more aggressive rehabilitation protocol was not allowed for patients after surgery.

### Limitations

Several limitations should be addressed to this study. First, though porcine bone and bovine tendons have been frequently considered similar to human knees with regard to BMD and biomechanical properties [14, 20], it is difficult to completely imitate the complex knee environment of human [37]. Second, it should be noted that, we only assess the influence of the interference screw on the ACL graft itself and does not evaluate the fixation strength to which the ACL is subjected in vivo, as previous studies had reported the effects of suturing the grafts on pull-out strength of tibia–graft constructs. Third, the biomechanical test on the grafts in vitro may be different with the forces on the graft during knee movement. Fourth, graft laceration does not only occur with the insertion of an interference screw, but also with repeated abrasion during cyclic loading. Our study did not perform did not perform a cyclic test to investigate the effect of graft abrasion. Despite the above limitations, our results may help to understand whether sutures reduced the graft laceration during tibial fixation. In the clinical practice, whipstitching the grafts does not decrease the tendon damage from interference screws.

## Conclusions

Using an in vitro model, our biomechanical study suggested that the ultimate failure load of grafts was reduced of approximately 25 % after one single insertion of a PEEK interference screw in ACL reconstruction. Suturing the ends of the grafts using different sutures (absorbable, Ethibond and UHMWPE sutures) did not decrease the graft laceration caused by interference screws.

## Data Availability

The data and materials in this paper are available from the corresponding author on reasonable request.

## Abbreviations

ACL: Anterior cruciate ligament reconstruction
UHMWPE: Ultra-high molecular weight polyethylene
PEEK: Poly-ether-ether-ketone
BMD: Bone mineral density
ANOVA: One-way analysis of variance
